# PhytoNanotechnology: Enhancing Delivery of Plant Based Anti-cancer Drugs

**DOI:** 10.3389/fphar.2017.01002

**Published:** 2018-02-09

**Authors:** Tabassum Khan, Pranav Gurav

**Affiliations:** ^1^Pharmaceutical Chemistry and Quality Assurance, SVKM's Dr. Bhanuben Nanavati College of Pharmacy, Mumbai, India; ^2^Quality Assurance, Alkem Laboratories Ltd., Mumbai, India

**Keywords:** phytoconstituents, anti-cancer, nanotechnology, selective targeting, drug delivery systems

## Abstract

Natural resources continue to be an invaluable source of new, novel chemical entities of therapeutic utility due to the vast structural diversity observed in them. The quest for new and better drugs has witnessed an upsurge in exploring and harnessing nature especially for discovery of antimicrobial, antidiabetic, and anticancer agents. Nature has historically provide us with potent anticancer agents which include vinca alkaloids [vincristine (VCR), vinblastine, vindesine, vinorelbine], taxanes [paclitaxel (PTX), docetaxel], podophyllotoxin and its derivatives [etoposide (ETP), teniposide], camptothecin (CPT) and its derivatives (topotecan, irinotecan), anthracyclines (doxorubicin, daunorubicin, epirubicin, idarubicin), and others. In fact, half of all the anti-cancer drugs approved internationally are either natural products or their derivatives and were developed on the basis of knowledge gained from small molecules or macromolecules that exist in nature. Three new anti-cancer drugs introduced in 2007, viz. trabectedin, epothilone derivative ixabepilone, and temsirolimus were obtained from microbial sources. Selective drug targeting is the need of the current therapeutic regimens for increased activity on cancer cells and reduced toxicity to normal cells. Nanotechnology driven modified drugs and drug delivery systems are being developed and introduced in the market for better cancer treatment and management with good results. The use of nanoparticulate drug carriers can resolve many challenges in drug delivery to the cancer cells that includes: improving drug solubility and stability, extending drug half-lives in the blood, reducing adverse effects in non-target organs, and concentrating drugs at the disease site. This review discusses the scientific ventures and explorations involving application of nanotechnology to some selected plant derived molecules. It presents a comprehensive review of formulation strategies of phytoconstituents in development of novel delivery systems like liposomes, functionalized nanoparticles (NPs), application of polymer conjugates, as illustrated in the graphical abstract along with their advantages over conventional drug delivery systems supported by enhanced biological activity in *in vitro* and *in vivo* anticancer assays.

**Graphical Abstract:**
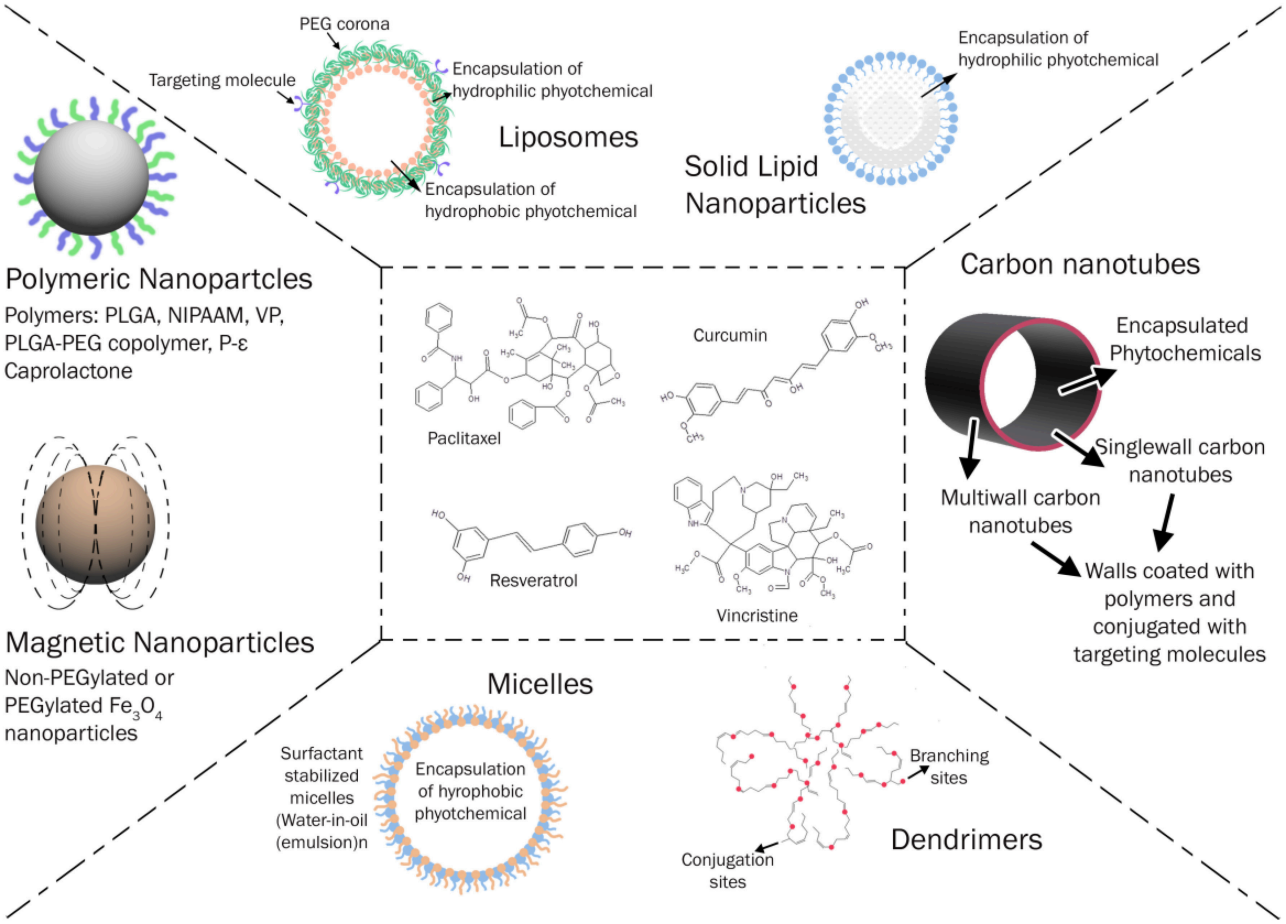
Versatile Drug delivery systems for anti-cancer Phytochemicals.

## Introduction

Cancer is a major public health issue and one of the most common causes of morbidity and mortality worldwide and the second leading cause of death globally. It was responsible for 8.8 million deaths in 2015. Approximately 70% of deaths of cancer occur in low and middle-income countries. The annual number of new cases is projected to rise from 14.1 million in 2012 to 21.6 million in 2030. In addition, the economic impact of cancer is significant; in 2010, the total annual economic cost of cancer was estimated at approximately US$ 1.16 trillion, threatening economies at all income levels as well as causing financial catastrophe for individuals and families. One defining feature of cancer is the rapid creation of abnormal cells that grow beyond their usual boundaries and which can then invade adjoining parts of the body and spread to other organs (metastases) which are a major cause of death from cancer (WHO Cancer Factsheet Feb, 2010).

Plants have been historically used in the alleviation of many diseases including cancer with over 60% of currently used anti-cancer drugs derived from natural sources. Nature is an attractive source of new therapeutic entities with plants, animals, marine organisms, and microorganisms all contributing to drugs with potential application as anti-cancer agents. Naturally occurring anticancer agents include vinca alkaloids, taxanes and its analogs, podophyllotoxin and its derivatives, camptothecin (CPT) and its derivatives, anthracyclines, and many others. In fact, half of all the anti-cancer drugs approved internationally are either natural products or their derivatives and were developed on the basis of knowledge gained from small molecules or macromolecules that exist in nature (Bhanot et al., 2011; Song et al., 2014).

Supplementary Table 1 represents the structural diversity of the anticancer agents obtained from natural resources.

Most of the clinically used anticancer drugs (formulated as conventional delivery systems) possess many significant limitations. These include their low solubility in water, unsuitability for oral administration, short half-life in the body, poor specificity associated with severe side effects (Cho et al., 2008). The therapeutic regimen is associated with severe systemic toxicity and development of multidrug resistance (MDR) primarily mediated by overexpression of proteins of the ATP binding cassette (ABC) transporter superfamily. These MDR proteins especially P-glycoprotein (Pgp) are responsible for energy dependent efflux of drugs, resulting in a reduced amount of chemotherapeutic agent present in cancer cells. Several Pgp inhibitors have been studied over the last few decades to overcome MDR in cancer. The first generation of ABC blockers [verapamil (VRP), cyclosporine A, and quinidine] are the most widely studied as these are already approved for other uses and can be repositioned and be clinically evaluated for new therapeutic use. Clinical studies on breast cancer have indicated that a combination of VCR and Pgp blocker VRP can enhance antitumor activity. This highlights the need to develop safe, effective, and novel drug delivery systems to target with MDR breast cancer (Taylor et al., 1997). Similar studies can be conducted on many anti-cancer phytochemicals in combination with Pgp blockers to improve their clinical efficacy in MDR cancer cell lines (Granja et al., 2016).

Another important factor is the tumor microenvironment that contributes to the development of MDR and affects a patient response to treatment. Nanocarriers offer promising delivery solutions for combination anticancer therapy that is required for treatment of MDR cancers. The benefits of nanocarriers include—their amenability to being engineered to achieve multiple effects using a single system, they improve the therapeutic index of drugs and can positively modulate their pharmacokinetic profile, they preferentially accumulate in the tumor microenvironment via EPR effect, their capacity to be conjugated to targeting moieties, and circumventing drug efflux by preferentially localizing agents in the perinuclear region of cell, away from membrane localized efflux pumps. These two drawbacks restrict the efficacy of these drugs and serve as the driving force for designing novel delivery systems to reduce the side effects and improve the clinical efficacy of existing drugs. It has also fuelled augmented research activity into development of new analogs of existing nature derived drugs and intensified search for new compounds from natural resources along with application of nanotechnology approaches to improve the safety and efficacy profiles of these drugs (Maeda, 2010; Thanki et al., 2013).

Nanotechnology has the potential to revolutionize cancer diagnosis and therapy. Often there is a defective, leaky vascular architecture associated with a tumor as a result of the poorly regulated nature of tumor angiogenesis. The interstitial fluid within a tumor is drained by a poorly formed lymphatic system. As a result submicron-sized particulate matter can easily extravasate into the tumor and be retained resulting in an “enhanced permeability and retention” (EPR) effect. This EPR effect is advantageous for a properly designed nanoparticulate system for passive targeting that allow nanocarriers loaded with cytotoxic agents to accumulate in the tumor tissues. Active targeting approaches acts by conjugating nanocarriers containing chemotherapeutics with molecules that bind to over-expressed antigens or receptors on the target cells. The biodistribution profile of nanocarriers can be fine-tuned by modifying their surface physico-chemical properties to target the tissue of interest, an outcome of recent advances in surface-engineering technology. This contributes to increased amount of drug reaching the targeted tumor sites with minimum systemic drug toxicity. Nanotechnologically modified drugs and drug delivery systems are increasing day by day in cancer treatment and some are used clinically with good results. Nanotechnology can be used for better cancer diagnosis, more efficient drug delivery to tumor cells, and molecular targeted cancer therapy that improving the therapeutic management of cancer patients (Mansoori et al., 2007; Schluep et al., 2009; Moorthi et al., 2011; Aliosmanoglu and Basaran, 2012).

The majority of current nanotechnology platforms for chemotherapy have involved repackaging of traditional anticancer agents into various forms of nanometer-sized delivery vehicles. Nanotechnology has provided a platform to improvise drug delivery using new concepts and carriers that conventional technologies have been unable to achieve. This review describes the application of nanotechnology in formulating drug delivery systems of some selected plant based anticancer agents [vincristine (VCR), paclitaxel (PTX), etoposide (ETP), curcumin (CUR), resveratrol, CPT, genistein, quercetin, and capsaicin (CAP) to name few of them] leading to an improved anticancer profile (Taratula et al., 2009).

**Figure 1:**
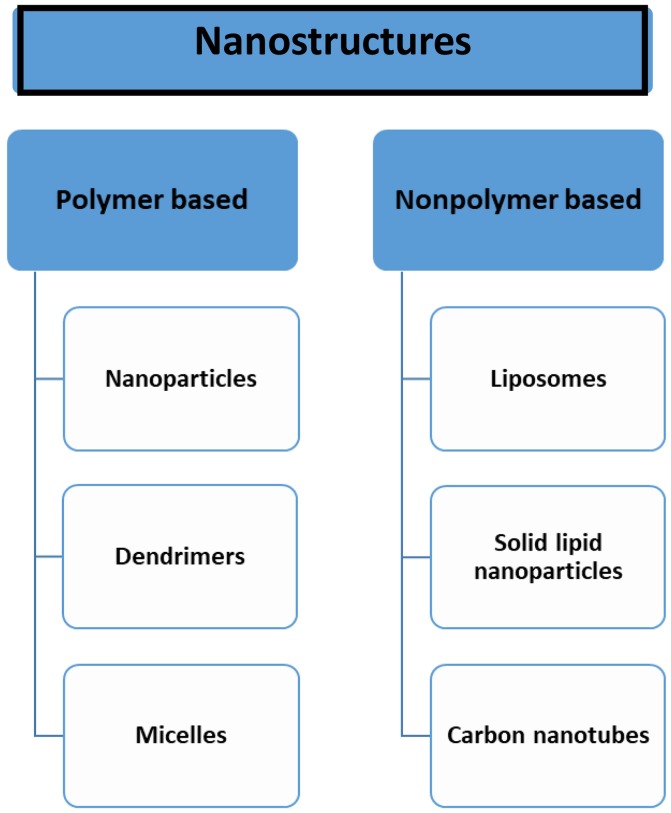
Nanotechnology based drug delivery vehicles.

Figure 1 depicts the nano-drug delivery vehicles used in formulation development and Supplementary Table 2 gives an overview of the various methods of preparation of nanodrug delivery systems used in development.

## Nanoparticles

The past two decades have witnessed many therapeutics based on nanoparticles (NPs) have been introduced in the market for therapeutic management of cancer. Advances in nanotechnology and an increased understanding of the importance of nanoparticle characteristics (size, shape, and surface properties) for biological interactions at the molecular level have created novel opportunities for development of NPs for versatile therapeutic applications.

Chen et al. prepared dual agent loaded—VCR and VRP poly(lactic-co-glycolic acid) (PLGA) NPs to reduce toxicity and enhance antitumor activity in multidrug resistant breast tumor Eenograft model. The PLGA NPs were prepared by emulsification/sonication method. They reported that the co-encapsulated NPs had lower toxicity (8.52 mg/kg) in comparison to VCR–VRP combination (4.93 mg/kg) and VCR–PLGANPs–VRP PLGANPs combination (7.85 mg/kg) in the acute toxicity study. The highest inhibition of tumor growth in the MCF-7/ADR (Adriamycin) human breast tumor xenograft was observed in the co-encapsulated NPs group (tumor mass = 0.32 g, inhibition efficiency = 64.04%) in comparison to VCR (tumor mass = 0.83 g, inhibition efficiency = 6.74%), VCR–VRP free combination (tumor mass = 0.62 g, inhibition efficiency = 30.34%), and VCR–PLGANPs plus VRP–PLGANPs combination (tumor mass = 0.47 g, inhibition efficiency = 47.19%). The *in vitro* and *in vivo* studies showcased that co-encapsulation of VCR and VRP into PLGA NPs at synergistic ratio exhibited good antitumor efficacy in MDR MCF-7/ADR human breast tumor xenograft models (Chen et al., 2014).

Das et al. loaded apigenin in PLGA nanoparticles (NAp) and studied its effect against ultraviolet B (UVB) and benzo(a)pyrene (BaP) induced skin tumor and mitochondrial dysfunction in mice. The results indicated superior effect for the Nap vs. apigenin alone attributed due to their smaller size, and faster mobility. The NAp reduced tissue damage and frequency of chromosomal aberrations, increased ROS accumulation to mediate mitochondrial apoptosis through modulation of several apoptotic markers and mitochondrial matrix swelling. The developed NAp showed ameliorative potential in combating skin cancer thereby showcasing better potential for use in therapeutic management of skin cancer (Das et al., 2013).

Wang et al. developed PTX and ETP co-loaded polymeric NPs and evaluated their cytotoxicity potential on MG63 and Saos-2 osteosarcoma cell lines. They used PLGA NPs to incorporate the two drugs by solvent evaporation method. The surface of these NPs was modified using polyethylene glycol (PEG) to prolong the blood circulation time. The co-encapsulated NPs exhibited a sustained release profile for PTX and ETP. *In vitro* cytotoxicity studies showed enhanced effect of combinational drug-loaded PLGA NPs (1.45 and 1.98 μg/ml) vs. the free drugs alone (PTX = 4.56 and 5.26 μg/ml; ETP = 6.12 and 7.15 μg/ml) and their combination (free ETP/PTX = 3.82 and 4.18 μg/ml) on MG63 and Saos-2 osteosarcoma cell lines owing to higher cellular uptake of NPs. The *in vitro* flow cytometry based cellular uptake studies indicated a time dependent cellular internalization of the NPs resulting in enhanced chemotherapeutic effect. The cell cycle progression studies indicated that the co-encapsulated PTX–ETP PLGA NPs arrested maximum number of cells at the same concentration as individual free drugs, the apoptosis fraction being almost double than the individual drugs in the early and late apoptosis phases of the cycle. These PTX–ETP PLGA NPs exhibited synergistic activity against osteosarcoma cancer cells (Wang et al., 2015).

Siddiqui et al. prepared PEG-PLA NPs of epigallocatechin-3-gallate (EGCG) recently and evaluated them on Mel 928 human melanoma cells (*in vitro* and *in vivo* studies). The results indicated an 8-fold increase in efficacy for the NPs vs. free EGCG. The *in vivo* studies indicated cell cycle phase arrest with modulation in the level of cyclins D1 and D3 protein expression for the EGCG NPs (Siddiqui et al., 2014).

Callewaert et al. developed surface modified NPs of ETP parenteral injectable solution (Teva®), loaded into PLGA or PLGA/P188-blended NPs using nanoprecipitation technique. The results of cytotoxicity studies conducted on murine C6 and F98 cell lines (glioblastoma) indicated that the use of PLGA and PLGA/P188 nanoencapsulation significantly enhanced the antitumor efficiency of ETP and gave a viable parenteral ETP formulation that circumvented the drawbacks of conventional injectable formulation (Callewaert et al., 2013).

Aygül et al. formulated PTX loaded PLGA NPs by emulsification solvent diffusion method. Cytotoxicity study of the PTX PLGA NPs was conducted on Caco-2 cells using the standard MTT assay. The results indicated that these NPs exhibited a significant cytotoxic effect in comparison to PTX alone—cell viability of 80% for PTX PLGA NPs vs. 90% for PTX alone (Aygül et al., 2013).

Bisht et al. developed polymeric NP-encapsulated curcumin (CUR) using micellar aggregates of cross-linked and random copolymers of N-isopropylacrylamide (NIPAAM), with N-vinyl-2-pyrrolidone (VP) and poly(ethyleneglycol)monoacrylate (PEG-A) that had a hydrophobic core and a hydrophilic shell. CUR has a major drawback of low oral bioavailability as demonstrated in many Phase I trials of CUR. Hence development nanotechnology based delivery systems that enable parenteral administration of CUR could potentially enhance the efficacy of CUR. This nanoCUR demonstrated comparable *in vitro* therapeutic efficacy to free CUR against human pancreatic cancer cell lines (BxPC3, AsPC1, MiaPaca, XPA-1, XPA-2, PL-11, PL-12, PL-18, PK-9, and Panc 2.03) as assessed by cell viability MTT assay. The results of cell uptake studies using fluorescence microscopy demonstrated enhanced uptake of nanoformulation of CUR by the pancreatic cell lines. This nanoformulation was found to enhance the solubility profile of CUR as well (Bisht et al., 2007).

Sebak et al. prepared NPs of noscapine with human serum albumin (HSA) for targeted delivery and evaluated them on SK-BR-3 breast cancer cells using pH coacervation method. The results indicated that the noscapine NPs were significantly better than free noscapine in reducing the cell viability of SK-BR-3 (Sebak et al., 2010).

Tang et al. developed PTX-loaded NPs of star-shaped cholic acid-core PLA-TPGS copolymer by a modified nanoprecipitation method and evaluated them in *in vitro* and *in vivo* studies on breast cancer MCF-7 cell lines. The results indicated that the CA-PLA-TPGS NPs had higher antitumor efficacy than the PLA-TPGS NPs and PLGA NPs (both in *in vitro* and *in vivo* studies) on MCF-7 cell lines demonstrating that these star-shaped cholic acid-core PLA-TPGS block copolymer could be considered as a potentially promising and effective strategy for breast cancer treatment (Tang et al., 2013).

Majumdar et al. encapsulated luteolin into a polymeric NPs comprising of PLA-PEG and evaluated them on H292 lung cancer cells. The results indicated the nanoluteolin exhibited a higher antiproliferative activity against H292 cells with lower IC_50_-values than free luteolin (Majumdar et al., 2014).

Sanna et al. formulated polymeric NPs encapsulating resveratrol (RSV) as novel prototypes for prostate cancer. Nanosystems, composed of a biocompatible blend of poly(epsilon-caprolactone) (PCL) and poly(D,L-lactic-co-glycolic acid)poly(ethylene glycol) conjugate (PLGA-PEG-COOH), were prepared by a nanoprecipitation technique. Cellular uptake of NPs was then evaluated in prostate cancer cell lines DU-145, PC-3, and LNCaP using confocal fluorescence microscopy and anti-proliferative efficacy was assessed using MTT assay. The results revealed that NPs were efficiently taken up by the PCa cell lines. These NPs significantly improved the cytotoxicity compared to that of free RSV (IC50 of NPs = 16 vs. 28.4 μM of free RSV) on DU-145 cell line (IC50 of NPs = 18 vs. 50.7 μM of free RSV) on LNCaP cell line, and (IC50 of NPs = 35.5 vs. 47.4 μM of free RSV) on PC-3 cell line (Sanna et al., 2013).

The same group developed novel cationic chitosan (CS)- and anionic sodium alginate (Alg)-coated PLGA NPs loaded with RSV by nanoprecipitation technique. Several studies have also been reported on the use of nanotechnology to vehicle RSV. In particular, RSV incorporation into mPEG-poly(ε-caprolactone)-based NPs resulted in significantly higher cytotoxicity against malignant glioma cells compared to an equivalent dose of free biomolecule. Solid-lipid NPs loaded with RSV also contributed to effectiveness of RSV on decreasing cell proliferation and demonstrated potential benefits for prevention of skin cancer. Moreover, other authors investigated the effect of RSV incorporated in liposome on the proliferation and UVB protection of cells, in order to enhance the efficacy in the prevention and treatment of human skin disorders caused by excessive exposure to UV radiation. Among the polymeric materials, PLGA is a widely used copolymer approved by the FDA for various medical and pharmaceutical applications, such as drug delivery. The combination of PLGA as a hydrophobic polymer and many natural hydrophilic biopolymers like gelatin or sodium alginate provides advantages for both hydrophilic and the hydrophobic nanoparticulate systems. CS and Alg are two major naturally occurring polysaccharides with hydrophilic characteristics that have gained increasing interest in the biomedical field particularly in the drug-delivery area. CS is a polycationic polymer that has one amino group and two hydroxylic functionalities in the repeating glycosidic residue. The amount of cationic or anionic polyelectrolytes employed as surface modifiers resulted in differences in size, surface charge, encapsulation capacity, and *in vitro* release behavior of RSV-loaded nanoprototypes. Stability studies revealed that encapsulation provides significant protection against light-exposure degradation, thereby contributing to increase the protection of the encapsulated RSV. The translational potential of these novel nanovehicles warrants biological evaluation in several experimental models, e.g., focusing on prostate and skin cancer as target diseases (Sanna et al., 2012).

Merlina et al. prepared PLGA NPs of ferulic acid by double emulsion method and evaluated them on NCI-H460 cells, non-small-cell lung carcinoma cell lines. The results depicted an increased anticancer effect for ferulic acid NPs vs. free ferulic acid. The ferulic acid NPs induced cytotoxicity reflected an increase in the level of reactive oxygen species, DNA damage, altered mitochondrial transmembrane potential, and apoptotic morphological changes demonstrating overall improved efficacy of the nanodrug delivery system (Merlina et al., 2012).

Gupta et al. prepared CUR NPs using silk fibroin polymer and a covalently bonded blend of silk fibroin and chitosan (SFCS) by capillary-microdot technique and evaluated them on MCF-7 and MDA-MB-453 breast cancer cell lines. The results indicated that the silk fibroin based CUR NPs displayed a higher cellular uptake and enhanced reduction in cell viability than the SFCS NPs in both breast cancer cell lines. Hence silk fibroin could prove to be a better nanodrug delivery vehicle and its application can be extended to development of NPs of many more phytochemicals (Gupta et al., 2009).

### Magnetic nanoparticles

Magnetic nanoparticles (MNPs), in particular iron oxide (also called magnetite or Fe_3_O_4_) NPs and their multifunctionalized counterparts are an important class of nanoscale materials that have attracted great interest for their potential applications in drug delivery and disease diagnosis. Owing to the recent advances in synthesis and surface modification technologies, a variety of new potential applications have become feasible for this class of nanomaterials that may revolutionize current clinical diagnostic and therapeutic techniques. The well-developed surface chemistry of Fe_3_O_4_ MNPs allows loading of a wide range of functionalities, such as targeting ligands, imaging, and therapeutic features onto their surfaces. It is now possible to fine-tune the physical parameters of MNPs, such as size, shape, crystallinity, and magnetism. Furthermore, MNPs have the potential for replacement or modification of the coating materials post-synthesis allowing tailoring of the nanoparticle's surface charge, chemical groups, and overall size. Due to their unique physicochemical properties and ability to function at the cellular and molecular level of biological systems, MNPs are being actively investigated as the next generation of targeted drug delivery vehicle. The design of such drug delivery systems requires that the carriers be capable of selectively releasing their payloads at specific sites in the body and thereby treat disease deliberately without any harmful effect on the healthy tissues. In this regard, MNPs represent a promising option for selective drug targeting as they can be concentrated and held in position by means of an external magnetic field. This allows high dose drug-loads to be delivered to a desired target tissue while minimizing the exposure of healthy tissues to the side effects from highly toxic drugs, e.g., chemotherapeutic agents. In addition, preclinical and clinical studies have proven them to be safe and some formulations are now FDA approved for clinical imaging and drug delivery. Thus, fabrication of MNPs as drug conjugates has the potential to greatly benefit inflammatory disease and cancer treatments, and diagnostics (Verma et al., 2013; Ali et al., 2016).

Castillo et al. synthesized PEG coated CPT loaded iron oxide superparamagnetic NPs using an iron co-precipitation method under alkaline conditions and evaluated them on H460 lung cancer cell line. Superparamagnetic iron oxide NPs (SPION) are particularly promising as delivery systems due to their low toxicity and their ability to be used both in cancer diagnosis and therapy. CPT suffers from a reduced *in vivo* antitumor efficacy owing to its poor water-solubility and chemical instability. CPT derivatives with improved solubility and stability have been developed however their overall therapeutic impact is modest due to their lower activity when compared to CPT. They reported that surface modification of the NPs by PEG enabled higher payload of CPT. No significant difference in the cytotoxic activity was observed among the CPT loaded on either the PEGylated or bare magnetic NPs (% apoptotic cells 80% for both). However, a different behavior of PEGylated and non-PEGylated magnetic NPs can be expected due to longer circulation times of the PEGylated ones (Castillo et al., 2014).

Verma et al. developed magnetic core-shell NPs for aerosol delivery of quercetin (QUR) by nebulization and evaluated their cytotoxicity in lung cancer cell line A549. They coated the surface of the MNPs with PLGA to improve the dispersion of QUR (a poorly soluble drug) in aqueous medium, confer stability against oxidation and ensure biocompatibility of the delivery system. The biocompatibility of the MNPs was characterized by conducting *in vitro* and *in vivo* studies. The results indicated a significant reduction in the number of viable A549 cells for QUR-loaded PLGA-MNPs. This study provided proof of concept of the optimized nanoplatform technology of drug loaded nanoparticle delivery in lung cancer using aerosol formulation (Verma et al., 2013).

## Liposomes

Liposomes are nanosized lipid carriers formed by the self-assembling phospholipid molecules in an aqueous environment. As liposomes are made up of lipids they are rapidly absorbed in liver and taken up by macrophages thus decreasing their efficacy. This can be avoided by coating liposome lipid surface with ligands such as monosialoganglioside or by incorporating cholesterol, polyvinylpyrrolidone polyacrylamide lipids, glucuronic acid lipids, or phospholipid distearoylphosphatidylcholine (DSPC) into liposomes that increases their circulating time in body. When the liposomes are coated with monosialoganglioside they are called stealth liposomes. The size of these liposomes is about 100 nm. The other type of liposomes i.e., non-stealth liposomes prepared from high phase transition temperature phospholipids helps in increasing circulation times and also accumulate within tumor tissue despite high levels of liver uptake. This surface modification of liposomes improves duration of drug release and also improves targeting of the drug to its site of action along with increased circulation time. Stability of the liposomes can be increased by incorporating cholesterol into it. The concentration of cholesterol is a crucial factor as it regulates the membrane properties. The advantages of using liposomal drugs as opposed to free drugs are well-documented in the literature and include the ability to selectively deliver liposomes to the desired site in the body (Chadha et al., 2008; Mehrabi et al., 2016).

Lu et al. prepared folic acid conjugated PEGylated liposomes of VCR (FA-PEG-LS/VCR) for multidrug resistant cancer therapy by thin-film hydration and extrusion method and evaluated their cytotoxicity on KBv200 cells (multidrug resistant variant), a human epidermoid nasopharyngeal carcinoma cell line using *in vitro* MTT assay and *in vivo* antitumor efficacy studies (tumor growth inhibition and apoptosis assessment studies by TUNEL). The results indicated that the IC50 of the PEGylated folic acid conjugated VCR liposomes was 23.99 nM vs. 1.10 μM for free VCR and 363.08 nM for PEG-LS/VCR. The results of *in vivo* experiments indicated that the folic acid conjugation significantly strengthened the antitumor efficacy of the PEGylated liposomes of VCR and also showed a higher apoptosis index in the TUNEL assay (24.1 vs. 14.4% and 11.8% for free VCR (Lu et al., 2013).

Lopes et al. formulated pH-sensitive liposomes of ursolic acid using lipid hydration method and evaluated them on MDA-MB-231 breast cancer cells. The results indicated that the liposomes had better anticancer activity than free ursolic acid indicating the improved anticancer activity of the nano-liposomes (Lopes de Araujo et al., 2013).

Bomana et al. prepared liposomes of VCR for achieving liposome circulation longevity, drug retention characteristics and *in vivo* antitumor activity. They encapsulated VCR inside EPC/cholesterol and DSPC/cholesterol vesicles by pH-gradient driven drug uptake processes. Encapsulation of VCR in DSPC/cholesterol liposomes resulted in a 1.7- to 2.1-fold increase in the LD_50_-values compared to free VCR when administered intravenously in DBA-2J and CD-1 mice models. Administration of free and liposomal VCR at doses in the range between 0.5 and 3.0 mg/kg resulted in a significant increase in the mean survival times and in the percent increase in life span (% ILS)-values of DBN-2J mice bearing either P388 or L1210 peritoneal tumors. The activity of VCR against tumors in DBA/2J mice appeared to be closely correlated to the VCR circulation longevity in the formulation indicating the benefits of liposomes as drug delivery systems that improve the toxicity profile of VCR and help increase the accumulation of drug at tumor sites (Bomana et al., 1995).

Odeh et al. prepared thymoquinone liposomes and evaluated them on MCF-7 cancer cells and fibroblast cells. The results indicated that the liposomes suppressed the proliferation of MCF-7 cells and exerted very low toxicity on normal periodontal ligament fibroblasts (Odeh et al., 2012).

Many research groups formulated berberine liposomes using different lipid compositions and evaluated them on selected cancer cell lines. The results indicated enhanced cytotoxicity in comparison to free berberine (Wen et al., 2011; Lin et al., 2014; Sailor et al., 2015).

## Carbon nanotubes

Carbon nanotubes are long, thin cylinders made up of carbon. These are synthetic rods that are only half the width of DNA. These are large macromolecules that are unique for their size, shape, and remarkable physical properties. The carbon nanotubes are derived from Graphene. As in grapheme carbon atoms are arranged in sp_2_ bonded structure they form honeycomb like patterns. They are of two types single-wall carbon nanotubes (SWCNTs) that have single layer of graphene and multi-wall carbon nanotubes (MWCNTs) that have more than one well of graphene. MWCNTs consist of concentric cylinders with the regular periodic interlayer spacing with a hollow center. This central core has a spacing of around 0.34–0.39 nm. This inner diameter differs depending on the number of layers. The outer diameter of these nanotubes ranges from 20 to 30 nm. The tips of MWCNTs are usually closed and their ends are capped. A property of carbon nanotubes is that they absorb near-infrared light waves and pass harmlessly through cells. However, when a beam of near-infrared light falls on carbon nanotubes, the excitation of electrons in the nanotubes occurs as a result the excess energy is produced in the form of heat that leads to the thermal destruction of cancer cells *in-vivo*. The surface of cancer cells contains numerous of receptors for vitamins known as folate, thus the nanotubes coated with the folate molecules would be attracted to folate receptors of diseased cells. This treatment induces coagulative necrosis, a form of cell death that involves protein denaturation and membrane lysis. Use of MWCNTs enables ablation of tumors with low laser power (3 W/cm^2^) and very short treatment times with minimal local toxicity and no evidence of systemic toxicity (Popov, 2014).

Tian et al. synthesized and functionalized multi-walled carbon nanotubes (MWNTs) as anticancer drug carriers for loading PTX using folic acid as the targeting ligand. These functionalized MWNTs exhibited good aqueous solubility, biocompatibility, and high targeting ability as indicated by *in vitro* cytotoxicity studies performed on HeLa cell line using MTT assay. The results showed significant enhancement in the cytotoxic capability thus improving the antitumor activity of the drug. The f-MWNTs-PTX complexes exhibited efficient targeting intracellular delivery and enhanced antitumor activity. These studies reinforce the application of functionalized carbon nanotubes-based complexes as promising drug delivery platform for improved cancer therapy (Tian et al., 2011).

## Dendrimers

Dendrimers are spherical macromolecules having highly branched structure of large number of peripheral groups that aid in encapsulation of hydrophobic drug compounds. They consist of a central core, branching units and terminal functional groups. The environment of the nanocavities and solubilizing properties of these cavities depend on the central core. Liquid crystals show the combined properties of both liquid and solid states. They can be made to form different size and shapes, with alternative polar and non-polar layers which includes aqueous drug solutions. Because of their unique physical properties (like monodispersity, water solubility and encapsulation ability, these macromolecules are very helpful in production of drug delivery vehicles. The properties of dendrimers are dominated by the functional groups on the molecular surface; however, there are examples of dendrimers with internal functionality. Dendrimers have a well-defined nanoscale architecture and large internal volume make them an attractive option for drug delivery and other biomedical applications. Their systematic structural architecture. The unique properties of dendrimers, as compared to linear polymers, render them of interest for intracellular drug delivery system for cancer therapy. Dendritic encapsulation of functional molecules allows for the isolation of the active site, a structure which mimics that of active sites in biomaterials. Also, it is possible to make water-soluble dendrimers, unlike most polymers, by functionalizing their outer shell with charged species or other hydrophilic groups (Abdel-Rahman and Al-Abd, 2013).

Malar et al. prepared dendrosomal CAP nanoformulation by esterification process and evaluated them for *in vitro* anticancer activity on VERO, Hep 2, and MCF-7 cell lines. The results indicated that these dendrimers showed a significant cytotoxicity on VERO cell line with an IC_50_ of 1.25 μg/mL and on MCF-7 and HEp2 cell lines with an IC_50_ of 0.62 μg/mL (Malar and Bavanilathamuthiah, 2015).

Sharma et al. formulated dendrimers of gallic acid with polyamidoamine (PAMAM) using Tomalia's divergent growth method and evaluated them on MCF-7 breast cancer cells. These dendrimers provided a high degree of surface functionality and versatility for drug loading. The IC_50_-values indicated that the gallic acid-loaded PAMAM dendrimers were significantly better than the free gallic acid proving the benefit of dendrimers as a suitable nanotechnology platform enhanced cytotoxicity on MCF-7 breast cancer cells (Sharma et al., 2011).

## Micelles

Micelles are collection of amphiphilic surfactant molecule that spontaneously aggregate and forms a spherical vesicle in water (size range of several tens of nanometers). The inner core of micelle is hydrophobic, thus can help in incorporation of hydrophobic drugs which are then released by some drug delivery mechanism. Conventional micelles consist of hydrophilic head and a hydrophobic tail made of small molecules consisting of the hydrocarbon portion of long fatty acids. They are most of times used as carriers for hydrophobic drugs and can be administered directly into the circulation. The molecular weight of polymer micelles are often high thus enabling maximum storage in the tissue of solid cancers. Micelles enter the tumor tissue easily as compared to other tissues. The concentration of micelles is often one order higher than in the surrounding area. The drug can be dissolved in the hydrophobic micelle core, or are bound chemically on the biodegradable polymer carrier. The preparation of polymeric micelles is simple but controlling the rate of drug release from these polymeric micelles is a tedious job. So the surface modified micelles were prepared, the chemical bond on the surface helps to control drug release. The activation of the micelles occurs in the tumor tissue environment thus preventing the drug release in the blood while circulation thus decreasing the toxicity of drug to normal cells (Lu et al., 2013; Lu and Park, 2013).

Qiu et al. developed luteolin polymeric micelles with monomethoxy poly(ethylene glycol)-poly(3 caprolactone) (MPEG-PCL) by self-assembly method and evaluated them on C-26 colon carcinoma cells. They studied the pharmacokinetics of free luteolin and the luteolin MPEG-PCL micelles in rats, the results indicating a higher bioavailable concentration of luteolin for the luteolin MPEG-PCL micelles. The *in vitro* cytotoxicity studies reflected a lower IC_50_ for the MPEG-PCL luteolin micelles suggesting that encapsulation of luteolin into MPEG-PCL micelles can potentially enhance the bioavailability and cytotoxicity (Qiu et al., 2013).

Blanco et al. prepared PEG-PLGA polymeric micelles of β-lapachone using film sonication method and evaluated them in mice with subcutaneous A549 lung tumor. The results of biodistribution studies of in mice indicated prolonged blood circulation and higher concentration of β-lapachone. In addition, the *in vitro* administration of the micelles to LLC tumors led to DNA damage and PARP-1 hyperactivation (Blanco et al., 2010).

Kumari et al. synthesized of block copolymeric micelles, methoxy-poly(ethylene glycol)-poly(D/L-lactide) (mPEG-PLA) to encapsulate CUR and evaluated their cytotoxicity in murine cancer cells, B16F10 (melanoma) and human breast cancer, MDA-MB-231 cell lines. The results of cellular uptake studies (flow cytometry studies) indicated that the cellular uptake of CUR in CUR-mPEG-PLA formulation was higher than that of free CUR in both the cell lines. The cytotoxicity of CUR was higher in mPEG-PLA micelles indicating mPEG-PLA polymeric micelles to be an efficient nanocarrier for CUR (Kumari et al., 2016).

Dong et al. prepared self-assembled biodegradable star-shaped polymeric micelles of honokiol using monomethoxy poly(ethylene glycol) (MPEG) and poly(3-caprolactone) (PCL) by direct dissolution ultrasonication method and evaluated them on CT26 murine colon carcinoma cells. The results indicated the polymeric micelles had a significantly enhanced dose related cytotoxicity on the CT26 cells (Dong et al., 2010).

Wei et al. formulated honokiol micelles using poly(3-caprolactone)-poly(ethylene glycol)-poly(3-caprolactone) copolymer (PCEC) and evaluated them on A549 human lung adenocarcinoma cells. The results indicated comparable anti-proliferative effect of free honokiol and honokiol-copolymeric micelles on A549 cells and were comparable showcasing their application in treatment of lung cancer (Wei et al., 2009).

Many phytodrug-loaded polymeric micelles for anticancer therapy are under investigation in preclinical studies to improve drug efficacy. Some of these are in various phases of clinical trials for e.g., a block copolymer of PEG and polyglutamate (PGlu) conjugated with 7-ethyl-10-hydroxy-campothecin for breast cancer reemphasizing the existing gap for novel delivery systems and scope for new therapeutic modalities for cancer management (Oerlemans et al., 2010).

### Solid lipid nanoparticles

Solid lipid nanoparticles (SLNPs) are widely used as a nanocarrier system for many drugs. These particles have size ranging from 50 to 1,000 nm and are made up of lipids which is stable at room temperature and body temperature. The lipids used in preparation of SLNPs include lipid acids, mono-, di-, or triglycerides, glyceride mixtures, or waxes that are stabilized using biocompatible surfactants. Over other drug deliveries the SLNPs have advantages of physical stability, protection of labile drugs from degradation, controlled release and ease of preparation. Production of SLNPs is relatively cost efficient and amenable to large scale production. The storage and drug leakage problems are very less in SLNPs than in liposomes (Ekambaram et al., 2012).

The SLNPs prepared using biodegradable polymeric materials showed significant decrease in toxicity and acidity. Most of lipophilic compounds can be efficiently incorporated into SLNPs thus the encapsulation of cytotoxic compounds in SLNPs can prove to be a best for oral administration of the drugs. A number of SLNPs or SLN-based systems have been developed for delivery of cytotoxic drugs such as doxorubicin, idarubicin, PTX, camptothecan, 7-ethyl-10-hydroxy-20(S)-CPT, ETP, flurodooxyuridine (FudR) and retinoic acid, and cholesterylbutyrate (Wei et al., 2015).

Serpe et al. prepared PTX loaded SLNPs that showed almost similar cytotoxic compared to the equivalent amount of drug in free solution. The *in vivo* efficacy of PTX loaded SLNPs was compared with free drug formulation using murine breast cancer mice model. It was found that the group of animal treated with PTX loaded SLNPs had significantly smaller tumor size and lower percent inhibition (Serpe et al., 2004).

Xu et al. prepared SLNPs of silibinin containing TPGS and phosphatidylcholine by using thin film hydration method and evaluated on MDA-MB-231 breast cancer cells. The results demonstrated enhanced cellular uptake of SLNPs almost twice that of free silibinin. These were corroborated by similar results in cell viability, invasion and migration assays. An interesting observation made by them was the suppression of the invasive and migratory capabilities of MDA-MB-231 cells at a concentration of 20 mg/mL of SLNPs via downregulation of the MMP-9 and Snail pathways (Xu et al., 2013).

Shen et al. coated SLNPs with hyaluronic acid so as to enhance its anti-tumor activity. The PTX was used as anti-cancer agent. The PTX-SLNPs were prepared by film-ultrasonic method. These SLNPs were coated with hyaluronic acid as cancer stem cells shows presences of CD44 that binds specially to hyaluronic acid. The *in vitro* and *in vivo* cytotoxicity evaluation was performed on B16F10 melanoma cells and in mouse xenograft model, respectively. The results showed the efficient intracellular delivery of PTX and induced substantial apoptosis in CD44^+^ cells *in vitro*. These PTX loaded HA-SLNPs targeted the tumor bearing tissues and subsequently exhibited significant antitumor effect with a low dose of PTX as compared to the pure drug (Shen et al., 2015).

An exhaustive review of the nanotech platforms for delivering some phytoconstituents as liposomes, nanoemulsions, micelles, SLNPs, and nanolipid carriers (NLCs) has been discussed with remarkable increase in their anticancer activity, an outcome of nanotechnology advantages (Chuan et al., 2015). A detailed summary of the application of nanotechnology for delivery of combination chemotherapy with many advantages over conventional combination therapy is presented and they have described many phytodrug combinations with synthetic and nature derived anticancer drugs (PCT and ETP; TPT and VCR; VCR and QUR; PCT and DOX; CUR and DOX; PCT and CUR to mention some) that have been developed as NPs, liposomes, lipid-polymer hybrid NPs and micelles delivering enhanced therapeutic efficacy for the combination drugs (Chen et al., 2017). Table 1 gives the comparative summary of the various nanotech delivery systems.

**Table 1:** Comparison of different nanotech delivery carriers for selected phytochemicals.

**Type**	**Nanoparticles**	**Solid Lipid Nanoparticles**	**Liposomes**	**Carbon Nanotubes**	**Micelles**	**Dendrimers**
Advantages	Fairly easy preparation. Targeted and drug delivery. Due to their small size Nanoparticles penetrate small capillary and are taken up by the cell which allows for efficient drug accumulation at the target sites in the body. Good control over size and size distribution. Good protection of the encapsulated drug. Retention of drug at the active site. Longer clearance time. Increased therapeutic efficacy. Increased bioavailability. Dose proportionality. Stable dosage forms of drug which are either unstable or have unacceptably low bioavailability in non-nanoparticulate dosages forms. Increased surface area results in a faster dissolution of active agents in an aqueous environment. Faster dissolution generally equates with greater bioavailability. Smaller drug doses. Reduction in fed/fasted variability. Less toxicity.	Possibility of controlled drug release and drug targeting. Increased drug stability High drug payload Incorporation of lipophilic and hydrophilic drugs No biotoxicity of the carrier Avoidance of organic solvents No problems with respect to large scale production and sterilization Increased Bioavailability of entrapped bioactive compounds	Improvement and control over pharmacokinetics and pharmacodynamics. Decreased toxicity. Enhanced activity of drugs against intracellular pathogens. Liposomes can be made to be target selective. Enhanced activity against extracellular pathogens	High stability *in vivo* because of their mechanical properties Large surface area available for multiple functionalization Capacity to easily pass biological barriers leading to novel biocompatible delivery systems Unique electrical and conducting properties for the development of new devices for diagnostics Empty internal space for encapsulation and transport of therapeutic and imaging molecules Bulk production associated to low costs	Very small size (diameter $\frac{1}{4}$ 10e100 nm) High structural stability Large amount of drug loading High water solubility Low toxicity Incorporation of various chemical species	Due to stringent control during synthesis, they have lower polydispersity index. As the density of branches increases the outer most branches arrange themselves in the form of spheres surrounding a lower density core and outer surface density is more and most of the space remains hollow toward core. Thus, increase entrapment of drug entrapment. Dendrimers might show an enhanced permeability and retention effect (depending on their M.W.) that allows them to target tumor cells more effectively than small molecules. The advantage of dendrimers is that they can be synthesized and designed for specific applications.
Limitations	Extensive use of polyvinyl alcohol as a detergent—issues with toxicity. Limited targeting abilities. Discontinuation of therapy is not possible. Cytotoxicity. Pulmonary inflammation and pulmonary carcinogenicity. Alveolar inflammation. The disturbance of autonomic imbalance by nanoparticles having direct effect on heart and vascular function.	Particle growth. Unpredictable gelation tendency. Unexpected dynamics of polymeric transitions Sometimes burst release	Sterilization Short shelf life and stability Encapsulation efficacy Removal from circulation by the Reticulo-endothelial system (RES) Interactions of liposomes with cells	Insolubility of as-produced materials—functionalization is required for rendering the material compatible in physiological conditions Strong tendency to aggregate Limited data on tolerance by healthy tissues Extremely high variety of carbon nanotube types standardization difficult	Difficult polymer synthesis Immature drug-incorporation technology Slow extravasation Possible chronic liver toxicity due to slow metabolic process	Complex, multistep procedures involved in the synthesis and processing of dendrimer-based nanoparticles. The complex structure limited drug loading capacities and increased amounts of polymers to be injected to reach a therapeutic dose of drug. Excessive conjugation of drug and other molecules to the surface of dendrimers can also induce undesirable changes in the material properties and polydispersity. Problem related to biodistribution. Unpredictable correlation between *in vivo* efficacy in animal models and in humans
Phytochemicals incorporated	VCR, ETP, PTX, CUR, RSV, CPT, QUR, EGCG	CUR, PTX, CPT, ETP	VCR, BER, QUR, thymoquinone, ursolic acid, ETP, CUR, RSV	PTX, QUR	CUR, PTX	Capsaicin
References	Gu et al., 2007 Cho et al., 2008 Mishra et al., 2010 Lim et al., 2011 Zu et al., 2011 Pimple et al., 2012, Siu et al., 2012 Tang et al., 2013, Mallamma et al., 2014, Sundar et al., 2014, Siddiqui et al., 2015, Suryani and Ismail, 2015 Granja et al., 2016 Han et al., 2016 Sajan et al., 2016 Salar and Kumar, 2016, Thadakapally et al., 2016 Yang et al., 2016 Zhou et al., 2016	Wong et al., 2007 Ekambaram et al., 2012 Yassin et al., 2013 Abd-Allah et al., 2014	Chadha et al., 2008 Narayanan et al., 2009 Ramanaa et al., 2012 Venegas et al., 2012 Shah et al., 2014 Mehrabi et al., 2016	Li et al., 2011 Tian et al., 2011 Popov, 2014	Nakanishi et al., 2001 Husseini and Pitt, 2008 Maeda et al., 2009 Mourya et al., 2011 Lu et al., 2013 Powar and Sharma, 2016	Naha et al., 2010 Abdel-Rahman and Al-Abd, 2013 Baig et al., 2015 Malar and Bavanilathamuthiah, 2015 Yang et al., 2015

Zhang et al. prepared co-encapsulated micelles of β-lapachone and PTX using PEG-PLA copolymer and evaluated them on A549 lung cancer cells. These micelles showcased enhanced antiproliferative effect vs. the individual drug micelles (IC_50_ = 0.16 mM for the co-encapsulated micelles, IC_50_ = 4.5 mM for β-lapachone micelles and 0.32 mM for PTX micelles). A very useful synergistic effect was observed for the co-encapsulated two agents against lung cancer (Zhang et al., 2015).

## Conclusion

About 65% of anticancer drugs introduced over the last 25 years have been derived from the natural sources. Chemical synthesis (either partial or total) has played an important role in supplying these nature derived compounds in large quantities and in preparing their novel analogs. Many of these compounds suffer from low solubility and poor bioavailability; Nanotechnology offers the advantages of increased bioavailability, prolongation of drug circulation time, multiple drug loading, all contributing to improved efficacy, and decreased toxicity. These nanotech based therapeutic delivery systems have many advantages such as water solubility, lower toxicity, biocompatibility, and amenability of their surface to further modifications for related applications. There is immense scope for the nature derived molecules to be formulated into nanotechnology based drug delivery system targeting the tumor microenvironment to combat MDR as nanotechnology based combination drug formulations. There is an existing gap and research initiatives to synthesize more tumor targeted nanotherapeutic delivery systems with high quality and yield of cytotoxic agents obtained from natural resources could prove effective in the overall management of cancer. Novel nanoformulations containing a synergistic combination of plant based drugs along with synthetic drugs could prolong drug circulation times, provide coordinated drug release, a better efficacy to toxicity ratio that could lead them into clinical trials and eventually to the bedside. Efficient formulation targeting strategies and evaluation of the targeting efficiency of NPs, and conforming to international standards for their toxicology and biocompatibility could pave the way for clinically viable phytochemical based anticancer therapies.

## Author contributions

TK has conceived the idea, edited, and built the manuscript. PG has conducted literature review and collated the information and prepared the draft review article.

### Conflict of interest statement

The authors declare that the research was conducted in the absence of any commercial or financial relationships that could be construed as a potential conflict of interest.
