# Supplementary material for: PhytoNanotechnology: Enhancing Delivery of Plant Based Anti-cancer Drugs
Source: Front Pharmacol. 2018 Feb 9;8:1002. doi: 10.3389/fphar.2017.01002 (PMC5811929; doi:10.3389/fphar.2017.01002)
Supplement: Supplementary file 1 [file Table1.docx]

**Table 1: Anticancer drugs obtained from natural resources**

| **Drug** | **Structure** | **Source** | **References** |
| --- | --- | --- | --- |
| Vincristine |  | *Catharanthus roseus* G. Don. (Apocynaceae). | Sisodiya et al., 2013  Cragg, et al., 2016 |
| Vinblastine |  |  |  |
| Paclitaxel |  | *Taxus brevifolia* | Sisodiya et al., 2013, Dhanikula et al, 1999 and Cragg, et al., 2016 |
| Docetaxel |  | Docetaxel is semi-synthetic analogue of paclitaxel. |  |
| Etoposide |  | It is a semi-synthetic derivative *Podophyllum peltatum* L. | Sisodiya et al., 2013, Zu et al., 2011 and Cragg, et al., 2016 |
| Camptothecin |  | *Camptotheca acuminata* |  |
| Irinotecan |  | Derviative of camptothecin | Sisodiya et al., 2013  Juillerat-Jeanneret et al., 2008 |
| Topotecan |  | Derivative of camptothecin |  |
| Aplidine |  | *Aplidium albicans* | Ruiz-Torres et al., 2017 and Bhanot et al., 2011 |
| Dactinomycin |  | *Streptomyces parvullus* | Bhanot et al., 2011 |
| Bleomycin |  | *Streptomyces verticillus* | Bhanot et al., 2011 |
| Doxorubicin |  | *Streptomyces peucetius* | Bhanot et al., 2011 |
| Curcumin  (Investigational) |  | *Curcumin longa* | Shanmugam et al., 2015, Thadakapally et al., 2016 |
| Resveratrol  (Investigational) |  | It is found in many plant species, including grapes, peanuts and various herbs | Cal et al., 2003, Zhou et al., 2016, Ferraz da Costa et al., 2017 and Siddiqui et al., 2015 |
| Genistein  (Investigational) |  | Isoflavonoid from soy products | Polkowski, K.,et al 2000 |
| Capsaicin |  | Active component of hot peppers of the genus Capsicum | Elkholi et al., 2014 and Cao et al., 2015 |
