# Supplementary material for: PhytoNanotechnology: Enhancing Delivery of Plant Based Anti-cancer Drugs
Source: Front Pharmacol. 2018 Feb 9;8:1002. doi: 10.3389/fphar.2017.01002 (PMC5811929; doi:10.3389/fphar.2017.01002)
Supplement: Supplementary file 2 [file Table2.docx]

**Table 2: Methods of preparation of nanoparticles**

| **Methods** | **Schematic representation** | **Drugs** | **Advantages** | **Disadvantages** | **References** | |
| --- | --- | --- | --- | --- | --- | --- |
| **Polymeric Nanoparticles** | | | | | | |
| Emulsion-solvent evaporation | 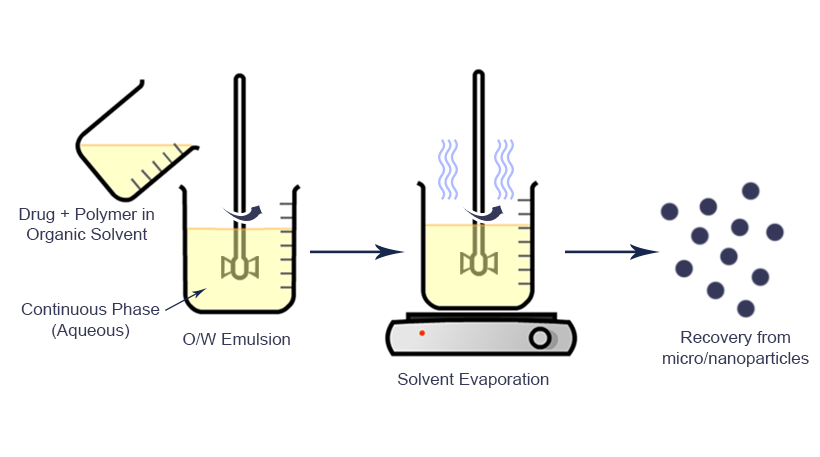 | VCR, PTX | - Simple and suitable for thermosensitive compounds. | - Low encapsulation efficiency especially for water soluble payloads. - Low yield and agglomeration of sticky particles. | Yang et al., 2016, | |
| Solvent displacement and interfacial deposition | 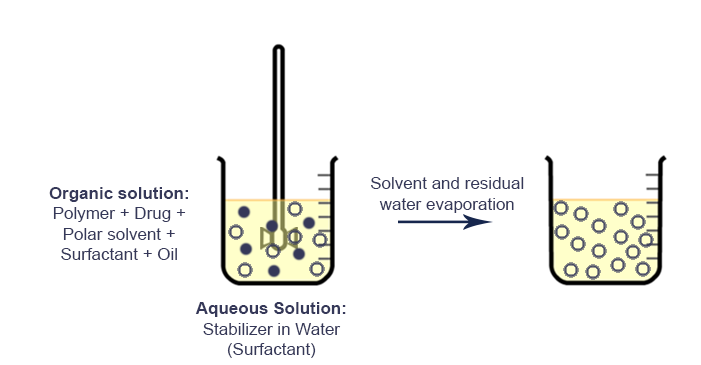 |  | - High-efficiency encapsulation. - The polymer is formed in situ, allowing the polymer membrane to follow the contours of the inner phase of an oil/water or water/oil emulsion | - The use of organic solvents is required for the external phase. - Washing of solvents and replacement by water represents a time consuming and difficult procedure. | Reis et al., 2006 | |
| Salting out method | 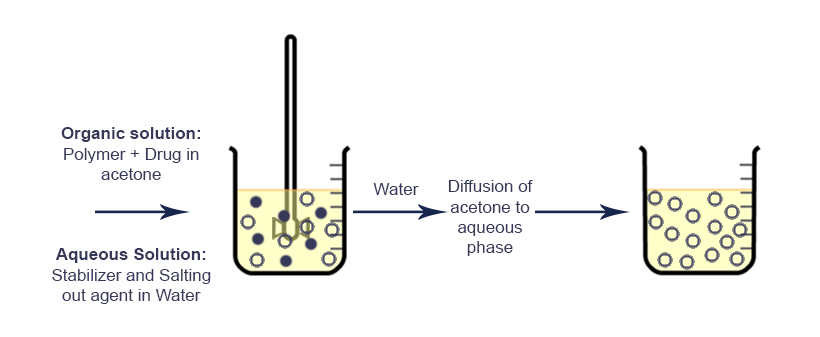 |  | - High efficiency and easy scale-up. - Minimizes stress to protein encapsulants. - Salting out does not require an increase of temperature, hence useful for thermosensitive drugs. | - Exclusive application to lipophilic drugs and the extensive nanoparticle washing steps. | Reis et al., 2006 | |
| Desolvation of macromolecules | 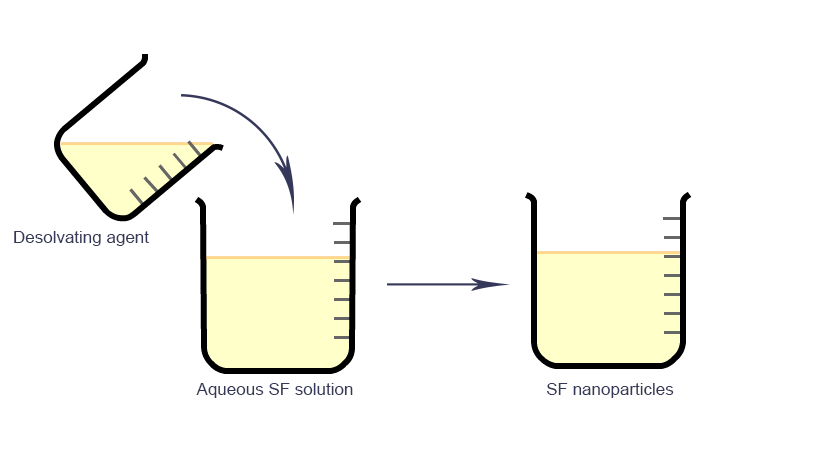 |  | - Capability of producing nanoparticles directly in aqueous suspension. | - The use of potentially toxic compounds such as glutaraldehyde and desolvating agents requires intensive purification. - Low yield. |  |  |
| - **Magnetic Nanoparticles** | | | | | | |
| Thermal decomposition | 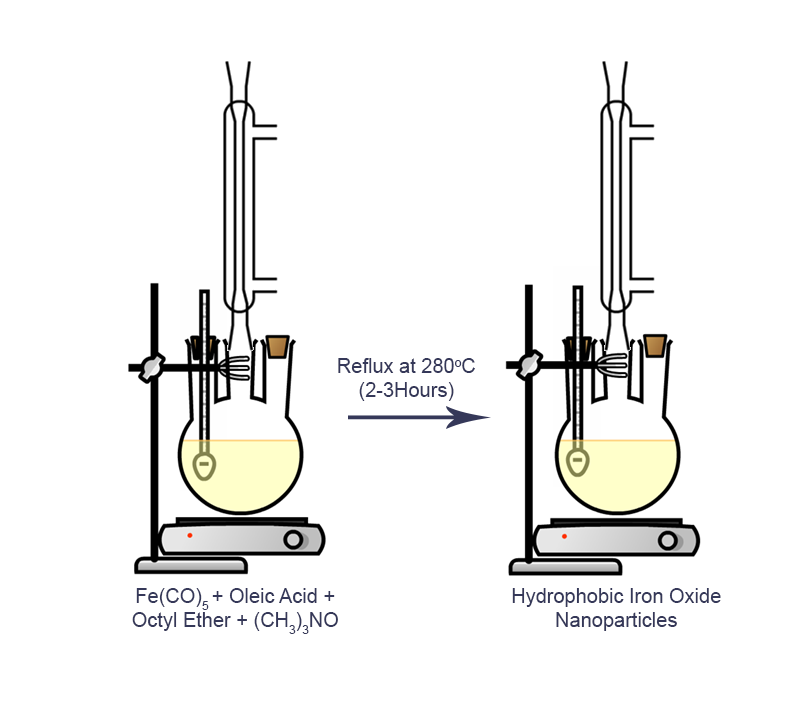 |  | - Facile control of product morphology, and good crystallization of products due to high reactivity of the reactants. - Ability to produce some metastable and unique condensed phases under high pressure conditions. | - Difficult to establish constant heating rate in the range where nucleation and growth occurs. | Sheng et al., 2014  Sundar et al., 2014 | |
| Sonochemical synthesis | 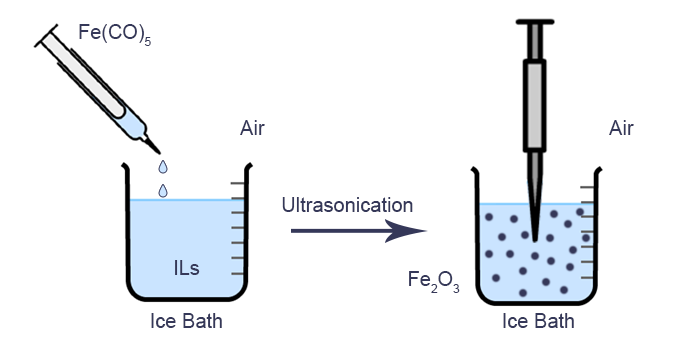 |  | - Ability to dissolve variety of materials with no measurable vapour pressure. - Simple, mild, highly selective and surfactant free and does not require any additional organic solvents, water or templating agent. - Effective shape control of the product. | - The mechanism is not well understood. | Badaini et al., 2011 and Ali et al., 2016 | |
| - **Liposomes** | | | | | | |
| Mechanical dispersion method | 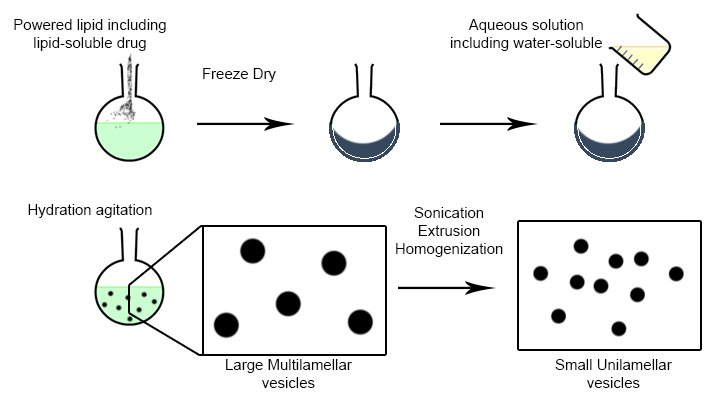 |  | - The resulting liposomes are larger in size. | - Difficult to attain required high temperature with small working volumes. | [Akbarzadeh](https://www.ncbi.nlm.nih.gov/pubmed/?term=Akbarzadeh%20A%5BAuthor%5D&cauthor=true&cauthor_uid=23432972)et al., 2013 | |
| Solvent dispersion method | 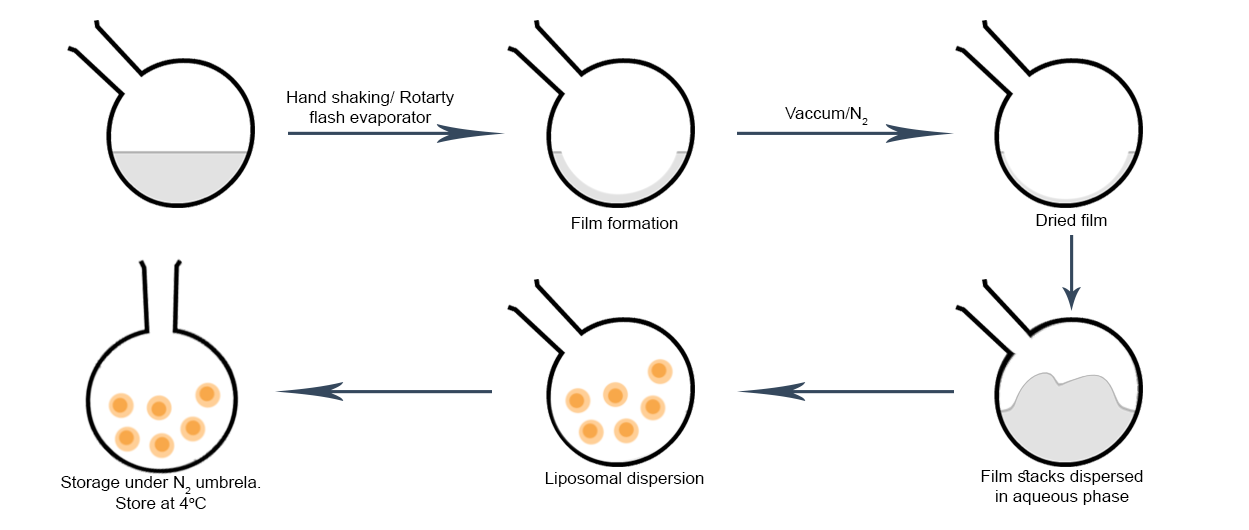 |  | - Simple process. | - The population is heterogeneous (30 to 110 nm). - The liposomes are very dilute and complete removal of ethanol is difficult because it forms into azeotrope with water, and the probability of the various biologically active macromolecules to inactivate in the presence of even low amounts of ethanol is high. | [Akbarzadeh](https://www.ncbi.nlm.nih.gov/pubmed/?term=Akbarzadeh%20A%5BAuthor%5D&cauthor=true&cauthor_uid=23432972)et al., 2013 | |
| - **Carbon Nanotubes** | | | | | | |
| Electric arc discharge | 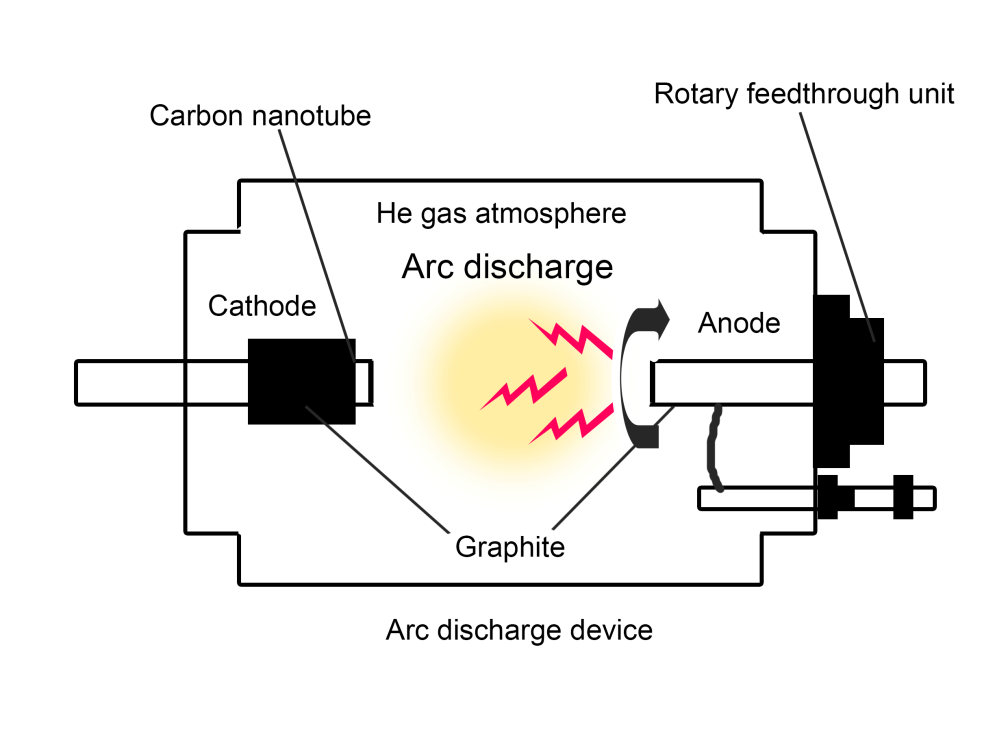 |  | - High yield of carbon nanotubes. | - A large amount of energy is needed for producing Arc. - High cost as it requires solid carbon/graphite target. | Rafique et al., 2011 | |
| Catalytic chemical vapour deposition | 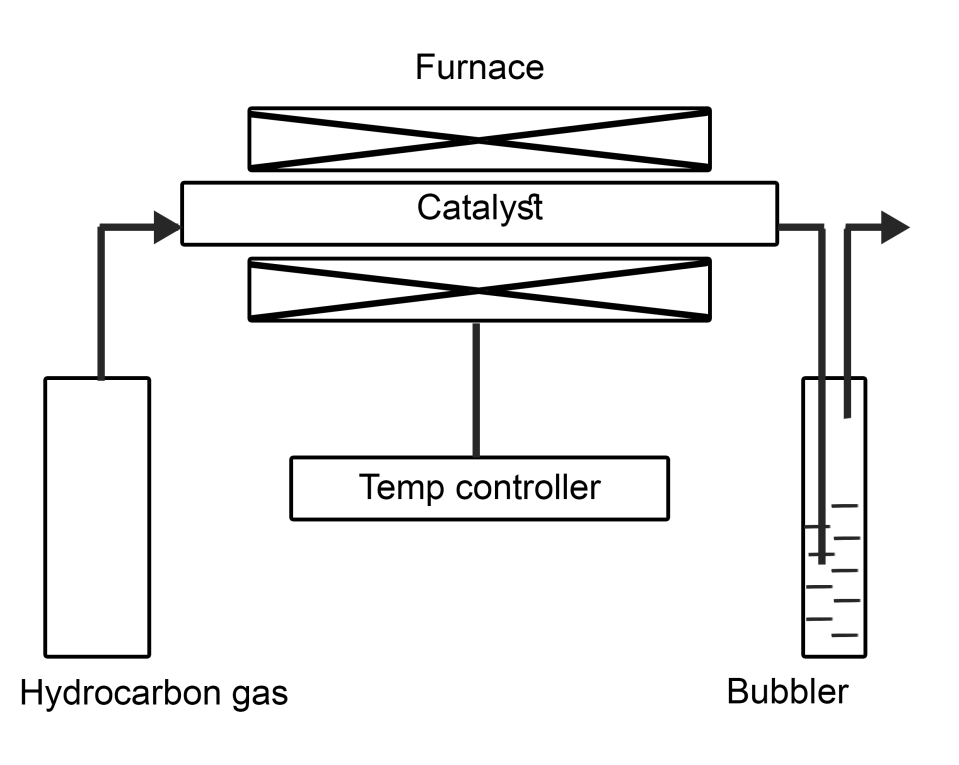 |  | - It is best-suited economic method for production of high purity single walled carbon nanotubes (SWNT) on large scale. | - Complexed System. | Rafique et al., 2011 | |
| Laser ablation | 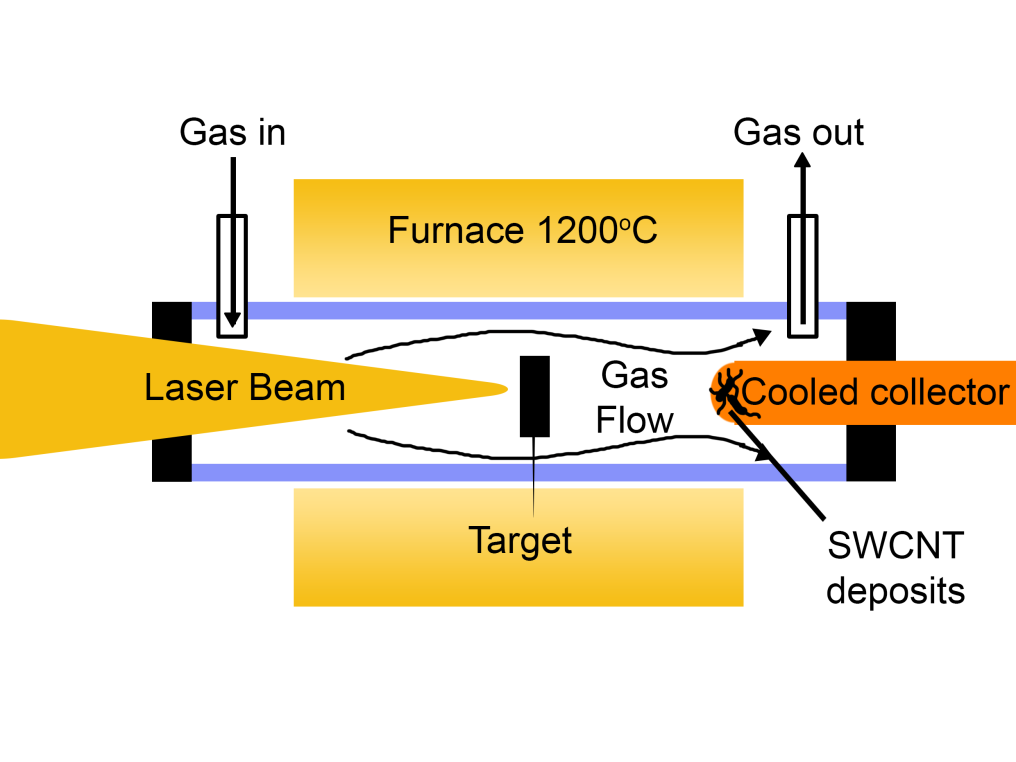 |  | - By varying temperature, catalyst composition and other process parameters average diameter and length of carbon naotubes can be varied. | - A large amount of energy is needed for producing laser ablation. - Expensive as it requires solid carbon/graphite target. - Growth of carbon nanotubes cannot be controlled. |  |  |
| - **Dendrimers** | | | | | | |
| Divergent method | 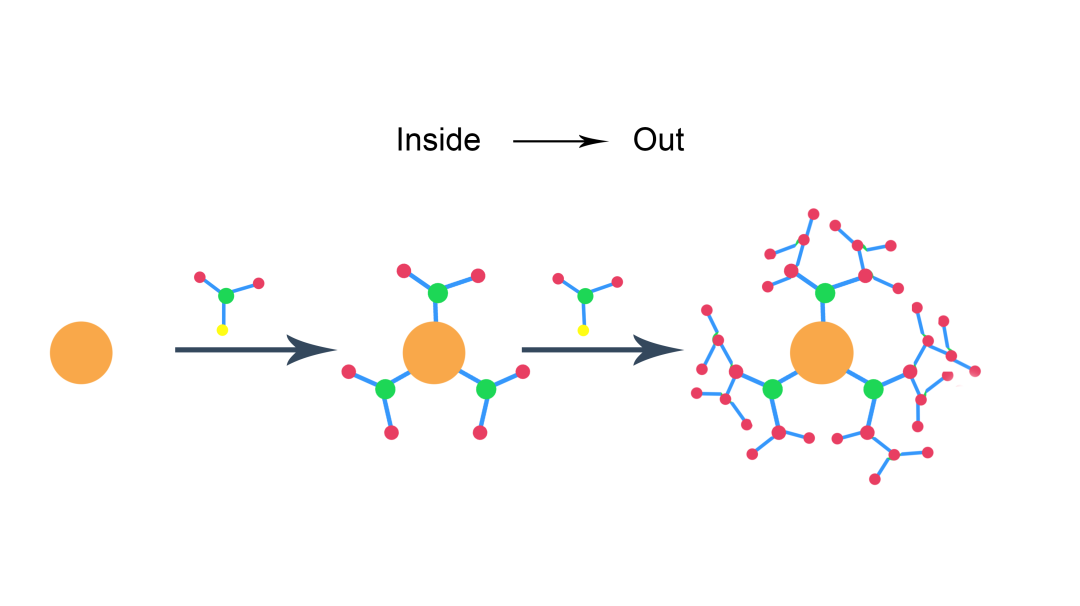 |  | - The dendrimer assembles to from a multifunctional core, which is extended outward by a sequence of reactions giving it versatility. | - The reaction must be determined to full completion to prevent mistakes in the dendrimer, which grounds trailing generations. - Difficult to purify because the relative size variation between perfect and imperfect dendrimers is very small. - Incomplete growth and side reactions lead to imperfect dendrimers. | Baig et al., 2015 | |
| Convergent method | 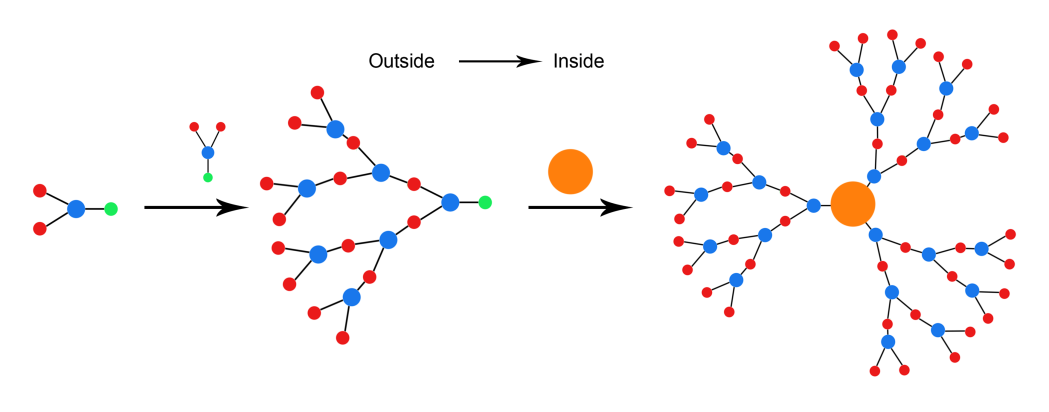 |  | - Easy to eliminate impurities and shorter twigs along the way, so that the final dendrimer is more mono-disperse. | - Not as large as those made by divergent methods because crowding due to steric property along the core is restrictive. |  |  |
| - **Micelles** | | | | | | |
| Direct dissolution | 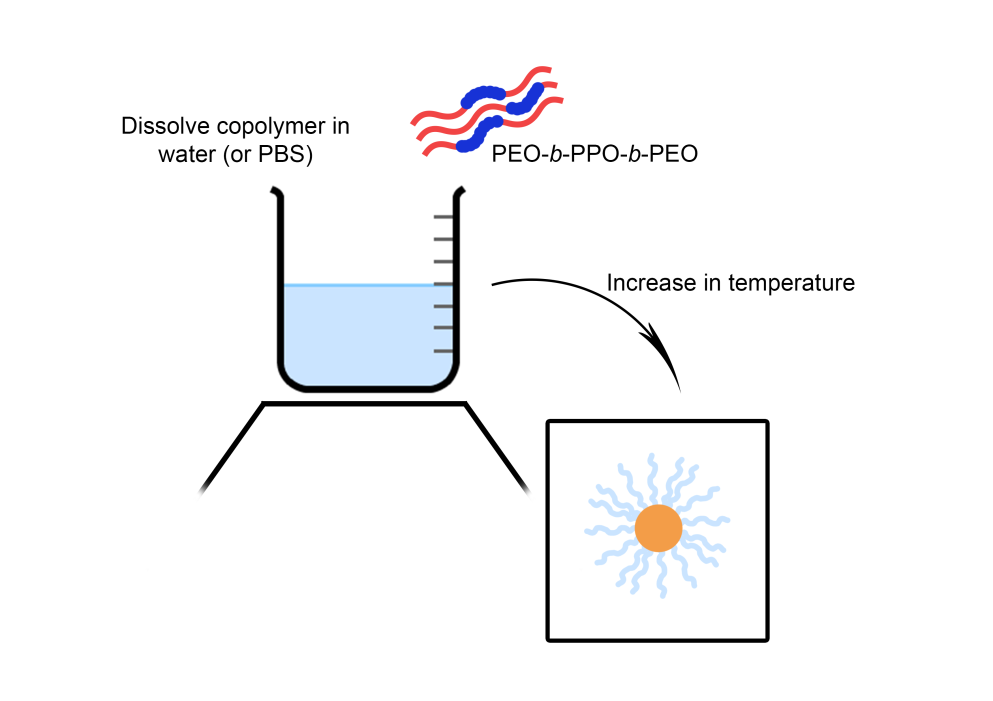 |  | - great potential as carrier of hydrophobic drugs. - liquid copolymers such as low molecular weight PEG-b-poly(CL-co-trimethylenecarbonate) can be easily mixed with hydrophobic drug in the absence of organic solvents to prepare micelles by direct dissolution | - preparation of polymeric micelles may not be possible for some polymers by direct dissolution in water at ambient temperature; - blood components may modify the extent of exchange between micelles and promote dissociation of polymeric micelles, even when they are administered far beyond the CMC | Kabanov et al., 2002, Powar et al., 2016 and Lu et al .,2013 | |
| Dialysis method | 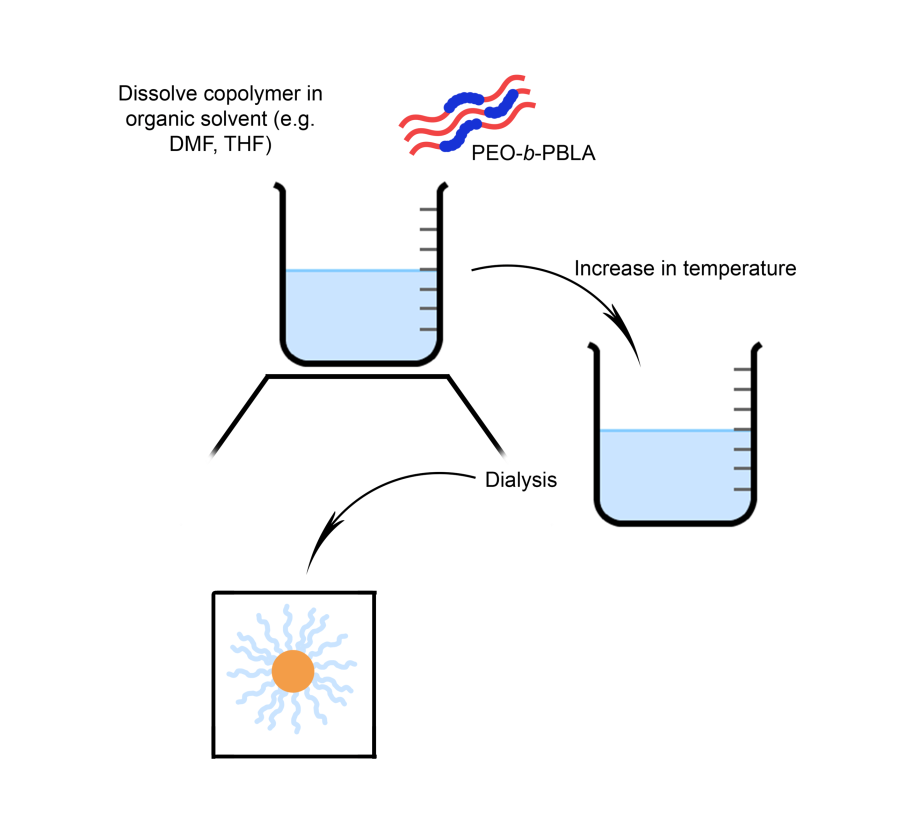 |  | - When the core forming blocks are long and more hydrophobic, this method can be useful. | - Chlorinated solvent used in the preparation is not safe. - Dialysis process often time consuming for getting efficient drug loading. | Mourya, V. K., et al. 2011 | |
| - **Solid Lipid Nanoparticles** | | | | | | |
| High pressure homogenization | 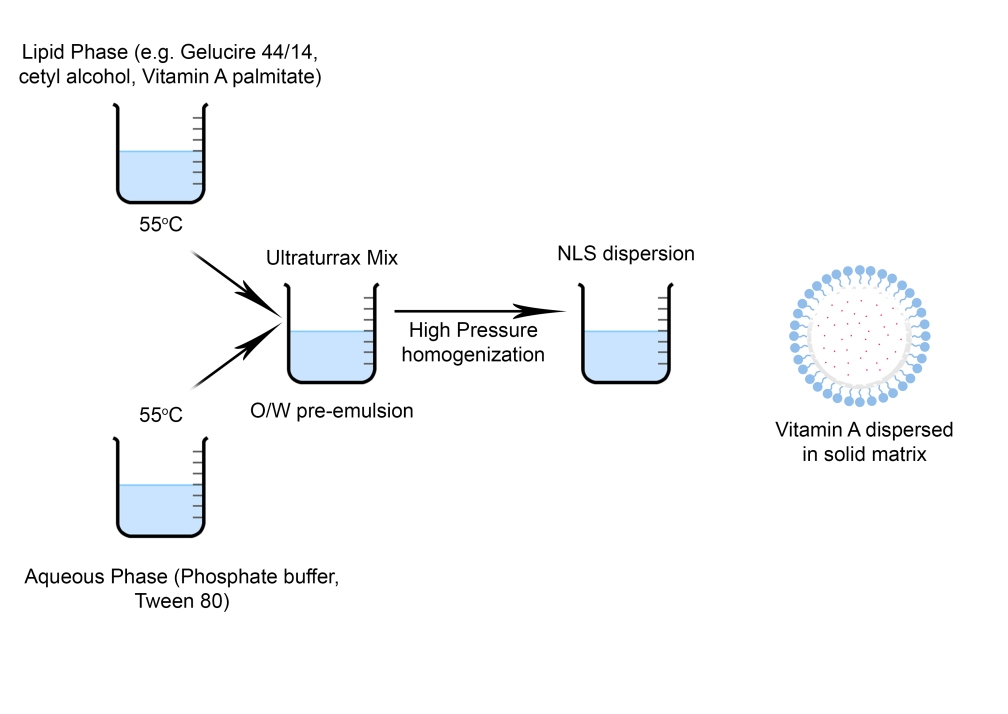 |  | - Large scale production of lipid nanoparticles are possible with this technique | - high energy conditions of temperature and pressure questioned its applicability in certain conditions | | Chaturvedi, S. P., et al. 2012 |
| Hot homogenization and cold homogenization | 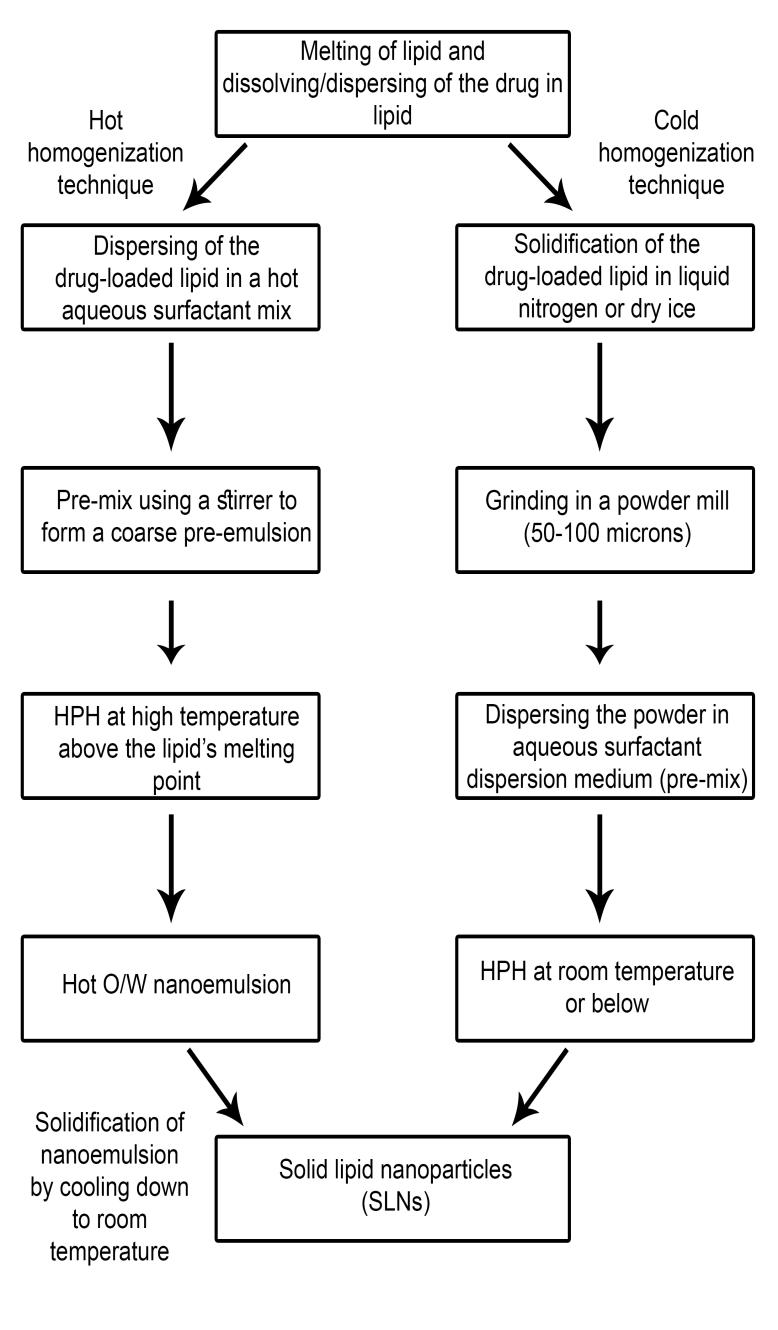 |  | - Hot Homogenization: - Used for the entrapment of lipophilic and insoluble drugs in the lipid. - Temperature sensitive compounds can also be processed by hot hph as exposure time to high temperatures is relatively short. - Cold Homogenization - Minimizes the thermal exposure to the drugs and active substances. Therefore, this technique may be applied for temperature sensitive compounds. Hydrophilic compounds can also be incorporated by this method which might partition from the liquid lipid phase to the water phase during the hot HPH. | - Hot Homogenization: - For hydrophilic drugs this procedure is not the most appropriated one. During the homogenization of the melted lipid phase the drug will partition to the water phase resulting in a too low encapsulation rate | | Chaturvedi, S. P., et al. 2012 |
| Solvent emulsification-diffusion Method | 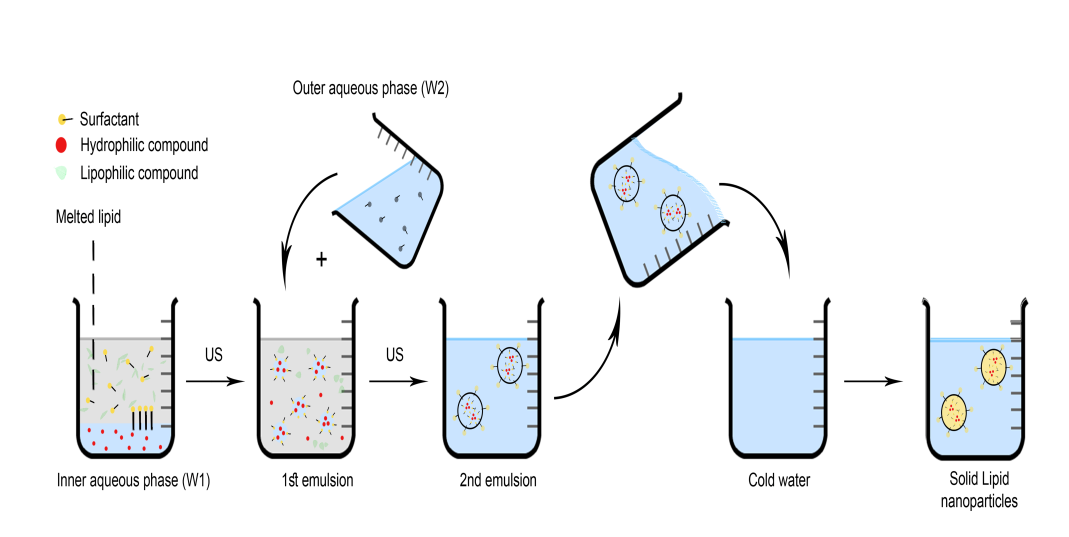 |  | - Particle size of SLN prepared by this method is small around 100 nm with narrow size distribution | The dispersions obtained in this procedure is fairly dilute similar to microemulsions method, and required to be concentrated by means of ultra-filtration or lyophilisation thus extra step is needed. | | Chaturvedi, S. P., et al. 2012 and Abd-Allah, F. I., et al. 2014 |
